# Before-and-after online community survey on knowledge and perception of COVID-19 pandemic

**DOI:** 10.1186/s12879-020-05602-6

**Published:** 2020-11-19

**Authors:** Wojciech Nazar, Julia Leszkowicz, Agata Pieńkowska, Michał Brzeziński, Agnieszka Szlagatys-Sidorkiewicz, Katarzyna Plata-Nazar

**Affiliations:** 1 Medical University of Gdańsk, Marii Skłodowskiej-Curie 3a, 80-210 Gdańsk, Poland; 2 Department of Pediatrics, Gastroenterology, Allergology and Nutrition, Medical University of Gdańsk, Nowe Ogrody 1-6, 80-803 Gdańsk, Poland

**Keywords:** Online survey, COVID-19, Medical personnel, Before-and-after study

## Abstract

**Background:**

COVID-19 pandemic impacts many communities worldwide. In this study the Poles’ knowledge about COVID-19 as well as people’s behaviours, attitudes and fears during the pandemic were assessed. Changes in these between the outset of the pandemic and the imposition of the strictest lockdown measures in Poland were investigated.

**Methods:**

Physicians, nurses, students of medicine-oriented faculties, non-medical professionals, students of non-medicine-oriented faculties and secondary school students were surveyed by an anonymous online questionnaire two times: at the onset of the pandemic and in the second week of the strictest lockdown. Statistical analyses were performed using non-parametric tests – Pearson Chi Square, Kruskal-Wallis tests.

**Results:**

In total 2618 responses were collected. At the beginning people knew that the respiratory system was attacked (97.9%); correctly identified the major symptoms of COVID-19 (95.0%) and ways to prevent infection: hand washing (99.8%), covering mouth (85.9%) and the need to call sanitary-epidemiological services if one experienced COVID-19-like symptoms (92.1%).

The biggest changes between the first and second phase of the study concerned behaviours: more people wearing facial masks (+ 37.5%) and staying at home (+ 66.1%). Respondents in the second wave of the survey were also more scared of the pandemic (+ 19.6%), economic crisis (+ 64.1%), and worried about their families (+ 26.5%). However, they were less afraid of the quarantine (lockdown) (− 18.2%). Nurses and physicians were the most worried groups.

**Conclusions:**

The study showed that even at the outset of the pandemic Polish population had a good initial knowledge about symptoms, transmission, and preventive behaviours regarding COVID-19. People revealed more short-term concerns, such as the worries about coping with quarantine and isolation. After a month, the knowledge and the concerns among the respondents changed. A shift towards long-term pandemic management issues was observed. Respondents reported to experience more fears concerning the pandemic in general, as well as economic and healthcare crises. Medical professionals reported higher level of fear of the pandemic than other groups included in this study. This study uses before-and-after approach which highlights the changes in people’s knowledge and perception of the COVID-19 pandemic during the pandemic’s progression.

**Supplementary Information:**

The online version contains supplementary material available at 10.1186/s12879-020-05602-6.

## Background

Coronavirus disease 19 (COVID-19) is the disease caused by the novel coronavirus 2019-nCoV (SARS-CoV-2). It is currently one of the biggest acute global threats to human health. The outbreak of the pandemic has affected many countries, causing problems concerning almost every aspect of everyday life. It has led to realignment in the world economy, deepened the medical crisis in many already vulnerable countries and it is currently the predominant topic covered by all media. The pandemic poses an unprecedented challenge for all health care systems worldwide. This is the first medical crisis in the recent history affecting different populations, countries and continents, requiring everybody to be aware and prepared for it.

The new coronavirus strain, discovered in December 2019 in the city of Wuhan in China [1], spread globally within few months. The first cases of COVID-19 in Europe were observed in January 2020 [2]. On March 4th, when the first case of COVID-19 was reported in Poland, there had already been more than 93,000 confirmed cases of infection and almost 3200 deaths reported globally [3]. As it was planned to distribute the questionnaires when the first case of COVID-19 in Poland will be observed, the first wave of the survey was launched on that day. The questions and answers of the survey were based on the current knowledge and prevailing atmosphere at that time, when many facts about the disease have not been discovered yet.

Within few days, the Polish government decided to implement precautions to stop the pandemic from spreading in Poland. Schools and universities were closed on March 12th [4]. Various public facilities and shops were closed on March 13th for a period of 2 weeks or longer. People were instructed to stay at home and refrain from travelling outside the country and the “state of epidemic threat” was declared nationally [5]. After a week, this official status was changed to the “state of epidemic” [6]. On April 1st, further restrictions amounting to a lockdown were introduced: leaving home was prohibited unless necessary, and only allowed individually or in groups of two. As a consequence of refusal to comply with the regulations a fine could be imposed [7].

The second wave of the survey began on April 9th, when there were about 5600 confirmed cases of infection and 174 deaths in Poland [8]. Globally, there were over 1,430,000 confirmed cases of infection and more than 85,500 deaths [9]. At the time the second phase of the study began, after nearly a month of lockdown, the Polish government had not abolished any of the restrictions yet.

The first aim of the study is to evaluate Poles’ knowledge about COVID-19 as well as people’s behaviours, attitudes and fears during the pandemic in Poland, depending on the socio-educational profile of the respondent. The second aim is to compare responses at the beginning of the epidemic before the lockdown restrictions and during its peak, at the time when the number of COVID-19 cases in Poland was very high and the restrictions imposed by the government had already affected life of the respondents.

To assess the level of knowledge and attitudes regarding SARS-CoV-2 and COVID-19 a short survey was created to evaluate the knowledge about coronavirus in the Polish population as a spontaneous reaction to the situation. The survey also covered social aspects of the pandemic, its influence on everyday life and peoples’ attitude to information disseminated in the media.

## Methods

### Materials

The survey was based on 2618 responses to the anonymous questionnaires, which were collected in two waves: at the outset of the epidemic and during the most severe COVID-19 lockdown in Poland. Demographic characteristics of the sample are presented in Table 1. Due to the pandemic conditions, it was decided to collect data online. The following cohorts were distinguished among the respondents: medicine-oriented community: physicians, nurses, medicine-oriented students, and non-medicine-oriented community: non-medical professionals, non-medicine-oriented students, secondary school students (over 18 years old). Such division was introduced, as physicians, nurses, and non-medical professionals are mentioned in other studies on COVID-19 perception [10–14]. Moreover, researches did not find many studies where the secondary or university students were asked about their knowledge and perception of the pandemic. These cohorts are also easily accessible for an online survey. The questionnaires were send via e-mails and posted on Facebook groups for medical professionals, students and non-medicine-oriented communities. Each group consisted of at least several thousand people, which aimed to reduce the selection bias. Respondents were surveyed to evaluate their knowledge regarding coronavirus and awareness of the threats of the epidemic outbreak in Poland. Although every resident of Poland was eligible for this survey, the biggest number of the respondents hailed from the northern regions of the country.

**Table 1:** Demographic characteristics of the sample, *n* = 2618

Cohort	Wave of study					
	First			Second		
	n	Mean age	Standard deviation	n	Mean age	Standard deviation
**Physician**	147	36.4	9.0	123	38.3	13.1
**Nurse**	68	39.1	12.2	108	39.4	10.8
**Student of medicine-oriented faculty**	287	22.0	2.3	713	21.8	2
**Non-medical professional**	168	37.2	13.3	277	45.4	10.4
**Student of non-medicine-oriented faculty**	174	21.6	1.9	128	21.9	2.1
**Secondary school student**	245	17.7	1.9	180	17.8	2.8
**All respondents (Mean)**	1089	26.4	10.8	1529	28.2	10.7

### Methods

The questionnaire was designed by the students of the Medical University of Gdańsk based on the WHO recommendations and information. Answers to the knowledge questions were based on the official WHO guidelines [15]. The survey consisted of twelve questions divided into four sections: demographics, knowledge, attitudes, and behaviours. The first section gathered data on the occupation, age, and the current place of residence of the respondent. The second part evaluated general knowledge about COVID-19 including its symptoms, routes of infection, and prevention methods. The third part measured the fear of the pandemic and which lockdown restrictions were the most difficult for people to cope with. The fourth part focused on sources of information on COVID-19. In the 4th question (infected body systems) a respondent could mark only one answer while the questions 5 to 11 (symptoms, transmission routes, prevention, what to do when experiencing symptoms, daily life difficulties, fears, knowledge sources) were multiple choice questions in which a respondent could pick more than one answer. The 12th question was a rating scale question. The research was conducted at two different phases of the epidemic in Poland. First questionnaires were collected in March (4/03/2020–11/03/2020), in the first week after the first case of COVID-19 had been announced in Poland. The survey was completed by 1089 respondents. On March 15th the Polish government announced the state of epidemic emergency and imposed numerous limitations on the entire society, so the first wave of data collection was terminated. The questionnaires for the second wave of the survey were collected in April (9/04/2020–16/04/2020) during the strictest COVID-19 lockdown in Poland, when the Polish society had been living in a restricted isolation for approximately a month. During the second phase, 1529 questionnaires were collected. The same questionnaire was used for both waves of the study (supplementary material). To increase the likelihood of obtaining a comparable sample, the survey was distributed by the same information channels in both rounds.

### Data analysis

Demographic data are presented in Table 1 as counts and (for age data) means with standard deviations. The percentage rates of answers were calculated for questions 4 to 11 for each of the six cohorts and for the whole sample (Tables 2, 4, and 5). For question 12 (how well-informed respondents felt), mean scores (on a 1–5 scale) were calculated (Table 3). All calculations were performed separately for both waves of the survey. All analyses of statistical significance were performed using non-parametric tests – Pearson Chi Square (questions 4–11) and Kruskal-Wallis tests (question 12). Threshold of statistical significance was set at *p* < 0.05. Statistical analysis was performed in Statsoft Statistica 13.3 software.

**Table 2:** Percentage rates of answers for questions of first phase surveys

Question	Answer	Rate of answers [%]							***p***-value
		Cohort							
		Physician	Nurse	Student of medicine-oriented faculty	Non-medical professional	Student of non-medicine-oriented faculty	Secondary school student	All respondents (Mean)	
**Which human organ system is usually infected by Coronavirus?**	respiratory system	100.0	100.0	99.0	97.0	97.1	95.9	97.9	0.1563
	nervous system	0.0	0.0	0.3	1.2	1.7	2.0	1.0	
	digestive system	0.0	0.0	0.3	1.2	0.0	0.8	0.5	
	muscular system	0.0	0.0	0.3	1.8	1.1	1.2	0.8	
	genitourinary system	0.0	0.0	0.0	0.0	0.0	0.0	0.0	
**What are the symptoms of Coronavirus disease 2019?**	fever	100.0	98.5	97.2	100.0	99.4	98.0	98.6	**0.0000**
	cough	98.0	98.5	97.2	95.8	96.6	96.3	96.9	
	breathlessness or breathing problems	98.6	98.5	95.5	94.6	87.4	90.6	93.6	
	muscle pain	72.8	60.3	62.4	66.7	56.3	55.9	61.9	
	fatigue	74.8	47.1	71.1	60.1	50.6	55.9	61.7	
**How does the virus spread to another person?**	by droplet transmission: coughing/sneezing	99.3	100.0	99.7	100.6	98.9	100.0	99.7	**0.0000**
	by indirect contact: through items touched by the infected person	57.8	45.6	43.6	43.5	37.4	39.6	43.7	
	by fecal-oral transmission: eating food contaminated with the virus	24.5	8.8	18.1	26.2	25.3	32.7	24.1	
	by direct contact: touching infected person	41.5	32.4	35.9	38.1	24.1	26.1	32.7	
**How to protect yourself from the virus?**	wear a standard (“surgical”) mask	5.4	5.9	6.3	4.8	5.7	9.8	6.6	**0.0000**
	wear a mask with a HEPA filter	48.3	26.5	39.7	19.0	25.9	42.9	35.4	
	wash your hands often	100.0	100.0	99.7	100.0	100.0	98.8	99.8	
	cover your mouth when coughing or sneezing	89.1	98.5	87.8	83.3	83.3	81.6	85.9	
	keep at least 1 m distance from others.	96.6	92.6	87.5	86.9	85.6	85.3	88.2	
	do not leave the house / flat	27.9	14.7	27.2	12.5	18.4	21.6	21.6	
	contact the sanitary and epidemiological station after you came back from another country where Coronavirus disease 2019 has been reported	70.7	88.2	77.0	60.1	82.2	85.3	77.0	
	drink alcohol	1.4	2.9	3.1	4.8	5.2	3.7	3.6	
	avoid products made in China	2.0	2.9	4.5	5.4	5.2	8.2	5.1	
**What should I do if signs of infection appear?**	notify the sanitary and epidemiological station	97.3	97.1	92.7	91.7	91.4	87.8	92.1	**0.0002**
	report to the emergency department in the nearest hospital	0.7	1.5	5.6	7.7	13.8	13.1	8.0	
	go to family doctor	0.7	2.9	3.5	9.5	8.0	10.6	6.3	
	report to an infectious disease ward or observation and infectious disease ward	74.8	76.5	72.1	56.5	70.1	70.2	69.6	
**What would be for you the biggest obstacle in everyday life due to Coronavirus disease 2019 pandemic?**	inability to go to your workplace or place of study	49.7	26.5	61.7	39.9	57.5	55.9	52.5	**0.0000**
	cancelled mass events, such as performances, conferences, concerts	13.6	2.9	32.8	20.2	31.0	47.3	29.4	
	cancelled trips, flights	32.7	14.7	37.3	39.3	30.5	53.9	38.2	
	quarantine or isolation from loved ones due to quarantine	73.5	86.8	58.9	76.8	73.6	64.9	69.1	
	no products and food in shops or higher prices in stores	52.4	54.4	62.4	54.2	71.8	64.5	61.2	
	no personal protection equipment in stock	44.9	50.0	32.8	34.5	29.3	31.0	34.8	
**Does any of the following aspects worries you due to the spreading infection?**	nothing worries me	10.2	5.9	32.1	27.4	21.3	28.6	24.2	**0.0000**
	my own health and life	26.5	42.6	11.1	25.0	26.4	17.1	21.1	
	health and life of my family	72.8	80.9	53.3	58.9	62.6	54.3	60.2	
	being quarantined	34.7	41.2	23.3	25.0	31.6	22.0	27.3	
	economic crisis	4.6	5.6	7.9	8.1	3.2	9.9	7.0	
	my education	4.5	1.9	2.2	3.3	5.1	4.5	3.6	
**Which of the following are your sources of knowledge about Coronavirus disease 2019?**	TV news	32.0	47.1	20.2	57.1	31.0	43.3	36.1	**0.0000**
	radio	13.6	23.5	8.7	31.5	14.9	22.4	17.9	
	newspapers	11.6	7.4	3.5	11.3	4.0	8.6	7.3	
	websites such as: onet, interia, wp, gazeta.pl, trójmiasto.pl	34.0	26.5	29.6	49.4	41.4	49.0	39.3	
	website of the Ministry of Health of the Republic of Poland	47.6	61.8	42.2	33.3	27.6	29.4	37.6	
	websites such as: onet.pl, interia.pl, wp.pl, gazeta.pl, trójmiasto.pl	87.1	66.2	75.3	36.3	46.6	50.6	60.1	
	family members and friends	14.3	7.4	35.9	31.0	33.9	41.6	31.4	
	social media: Facebook, Tweeter, Instagram	51.7	32.4	42.5	41.1	62.6	53.9	48.7

**Table 3:** Mean scores in answers to the 12th question: Do you feel sufficiently informed about the epidemic?

Wave of study	Cohort							
	Physician	Nurse	Student of medicine-oriented faculty	Non-medical professional	Student of non-medicine-oriented faculty	Secondary school student	All respondents (Mean)	***p***-value
**First**	3.4	3.6	3.6	3.7	3.3	3.4	3.5	**0.0003**
**Second**	3.8	3.4	3.7	3.6	3.7	3.7	3.7	0.0640
**Difference**	0.4	−0.2	0.1	−0.1	0.4	0.3	0.2	–

1 means “not sufficiently informed” and 5 means “sufficiently informed”

## Results

In total, 2618 surveys were collected, with about 80% of respondents residing in the Pomorskie (Pomeranian) voivodeship, Poland. Age and profession characteristics of the sample are presented in Table 1.

Most of the respondents knew that COVID-19 attacked the respiratory system (97.9%, Table 2) and that it was an airborne disease (99.7%). They also correctly identified the main symptoms of COVID-19: fever (98.6%), cough (96.9%), shortness of breath (93.6%). Additionally, the medicine-oriented sub-population knew about muscle pain and tiredness. Respondents also knew the main prevention measures including hand washing (99.8%), covering mouth (85.9%), and social distancing (88.2%), as well as the need to call the sanitary-epidemiological service (92.1%) or to visit an infectious diseases ward (69.6%) if one experienced COVID-19-like symptoms. A small percentage of studied population said they would also visit a general practitioner (6.3%) or an emergency department (8.0%). People were worried about quarantine (69.1%), grocery price increases (61.2%), and their families (60.2%). Most of the questioned felt well informed about the virus (3.5/5, Table 3). The main sources of knowledge for non-medicine-oriented respondents were general news websites or social media, while medicine-oriented community chose science-oriented or governmental websites more often.

When comparing the results from the first and second wave of the survey, more people reported wearing facial masks and staying at home (+ 37.5%, + 66.1%, Tables 4 and 5). Respondents were also more aware that they should not visit a general practitioner or an emergency department (1.6%, − 6.3%) if symptomatic; that the SARS-CoV-2 could spread via direct (+ 18.1%) or indirect contact (+ 9.2%) and that tiredness was a symptom of COVID-19 (+ 9.1%). People were more worried about the pandemic generally (+ 19.6%), economic (+ 64.1%) and education (+ 37.3%) crises, and about their families (86.8%, + 26.5%). However, they were less afraid of being quarantined (− 18.2%) or price increases (− 36.7%). Among nurses and physicians not a single respondent selected “not worried about anything” answer, while only these two populations were not concerned about being absent at work (Table 4). More respondents in the second phase of the survey reported visiting governmental and science-oriented websites (+ 27.4%, + 9.6%, respectively), especially among the non-medical section of the sample, and watching TV (+ 16.4%) to learn about COVID-19. Non-medical professionals and nurses were the only cohorts that felt slightly worse informed about the COVID-19 in the second wave of the survey. In other groups, information confidence increased (Table 5).

**Table 4:** Percentage rates of answers for questions of second phase surveys

Question	Answer	Rate of answers [%]							***p***-value
		Cohort							
		Physician	Nurse	Student of medicine-oriented faculty	Non-medical professional	Student of non-medicine-oriented faculty	Secondary school student	All respondents (Mean)	
**Which human organ system is usually infected by Coronavirus?**	respiratory system	100.0	100.0	100.0	100.0	100.0	98.9	99.9	0.1033
	nervous system	0.0	0.0	0.0	0.0	0.0	0.0	0.0	
	digestive system	0.0	0.0	0.0	0.0	0.0	0.0	0.0	
	muscular system	0.0	0.0	0.0	0.0	0.0	1.1	0.1	
	genitourinary system	0.0	0.0	0.0	0.0	0.0	0.0	0.0	
**What are the symptoms of Coronavirus disease 2019?**	fever	100.0	100.0	99.0	99.3	98.4	95.6	98.8	**0.0000**
	cough	100.0	97.2	95.4	97.8	93.8	89.4	95.5	
	breathlessness or breathing problems	100.0	100.0	99.7	99.6	99.2	98.3	99.5	
	muscle pain	82.1	72.2	57.5	71.1	57.8	57.2	63.0	
	fatigue	87.0	79.6	68.9	77.6	65.6	55.6	70.8	
**How does the virus spread to another person?**	by droplet transmission: coughing/sneezing	100.0	100.0	100.0	99.3	98.4	99.4	99.7	**0.0058**
	by indirect contact: through items touched by the infected person	72.4	64.8	57.1	66.1	67.2	61.1	61.8	
	by fecal-oral transmission: eating food contaminated with the virus	31.7	17.6	19.8	25.3	32.0	25.6	23.3	
	by direct contact: touching infected person	48.0	41.7	38.7	45.5	42.2	45.0	41.9	
**How to protect yourself from the virus?**	wear a standard (“surgical”) mask	63.4	52.8	47.4	56.0	57.0	47.2	51.4	**0.0000**
	wear a mask with a HEPA filter	83.7	76.9	73.4	70.0	68.0	68.9	72.9	
	wash your hands often	99.2	99.1	99.7	98.9	100.0	99.4	99.5	
	cover your mouth when coughing or sneezing	87.0	94.4	90.0	90.3	86.7	84.4	89.2	
	keep at least 1 m distance from others.	90.2	89.8	93.3	91.0	86.7	91.1	91.6	
	do not leave the house / flat	80.5	79.6	90.6	82.3	88.3	93.9	87.7	
	contact the sanitary and epidemiological station after you came back from another country where Coronavirus disease 2019 has been reported	76.4	92.6	92.1	83.4	88.3	86.1	88.3	
	drink alcohol	0.8	0.0	0.6	2.2	1.6	3.3	1.2	
	avoid products made in China	0.8	2.8	2.7	2.5	2.3	6.7	2.9	
**What should I do if signs of infection appear?**	notify the sanitary and epidemiological station	97.6	98.1	99.4	96.8	100.0	98.9	98.7	**0.0730**
	report to the emergency department in the nearest hospital	2.4	2.8	1.0	0.0	1.6	5.6	1.6	
	go to family doctor	1.6	2.8	1.1	4.0	0.8	4.4	2.2	
	report to an infectious disease ward or observation and infectious disease ward	63.4	75.9	46.1	50.2	43.8	48.9	50.5	
**What would be for you the biggest obstacle in everyday life due to Coronavirus disease 2019 pandemic?**	inability to go to your workplace or place of study	31.7	15.7	76.2	43.3	67.2	68.9	60.8	**0.0000**
	cancelled mass events, such as performances, conferences, concerts	15.4	9.3	30.2	16.2	35.2	50.6	27.8	
	cancelled trips, flights	39.0	19.4	32.4	38.6	36.7	37.8	34.1	
	quarantine or isolation from loved ones due to quarantine	77.2	71.3	73.9	73.3	70.3	82.8	74.6	
	no products and food in shops or higher prices in stores	12.2	28.7	27.9	21.3	28.1	19.4	24.5	
	no personal protection equipment in stock	56.9	61.1	39.4	42.6	33.6	22.2	40.4	
**Does any of the following aspects worries you due to the spreading infection?**	nothing worries me	0.0	0.0	4.9	4.3	6.3	8.9	4.6	**0.0000**
	my own health and life	28.5	46.3	21.9	35.7	25.0	22.8	27.0	
	health and life of my family	92.7	93.5	87.7	87.0	83.6	77.2	86.8	
	being quarantined	20.3	29.6	6.6	6.1	4.7	6.1	9.0	
	economic crisis	74.8	57.4	71.9	73.3	75.8	66.7	71.1	
	my education	22.8	11.1	53.6	23.1	46.9	43.9	40.9	
**Which of the following are your sources of knowledge about Coronavirus disease 2019?**	TV news	32.5	61.1	50.8	59.6	50.8	57.8	52.5	**0.0000**
	radio	10.6	24.1	15.0	23.1	15.6	24.4	17.9	
	newspapers	11.4	13.9	4.5	12.6	2.3	7.2	7.3	
	websites such as: onet, interia, wp, gazeta.pl, trójmiasto.pl	39.8	43.5	39.8	59.9	47.7	57.2	46.4	
	website of the Ministry of Health of the Republic of Poland	58.5	77.8	69.8	61.4	59.4	51.7	64.9	
	websites such as: onet.pl, interia.pl, wp.pl, gazeta.pl, trójmiasto.pl	91.1	75.0	75.6	58.8	64.8	49.4	69.8	
	family members and friends	16.3	13.0	32.4	24.2	41.4	55.0	31.7	
	social media: Facebook, Tweeter, Instagram	33.3	43.5	46.4	35.0	56.3	56.1	45.1

**Table 5:** Difference between percentage rates of answers for questions of first and second phase surveys

Question	Answer	Rate of answers [%]						
		Cohort						
		Physician	Nurse	Student of medicine-oriented faculty	Non-medical professional	Student of non-medicine-oriented faculty	Secondary school student	All respondents (Mean)
**Which human organ system is usually infected by Coronavirus?**	respiratory system	0.0	0.0	1.0	3.0	2.9	3.0	2.0
	nervous system	0.0	0.0	−0.3	−1.2	− 1.7	−2.0	− 1.0
	digestive system	0.0	0.0	−0.3	−1.2	0.0	−0.8	−0.5
	muscular system	0.0	0.0	−0.3	−1.8	− 1.1	− 0.1	− 0.7
	genitourinary system	0.0	0.0	0.0	0.0	0.0	0.0	0.0
**What are the symptoms of Coronavirus disease 2019?**	fever	0.0	1.5	1.8	−0.7	−1.0	− 2.4	0.1
	cough	2.0	−1.3	− 1.8	2.0	−2.8	−6.9	− 1.4
	breathlessness or breathing problems	1.4	1.5	4.2	5.0	11.9	7.7	6.0
	muscle pain	9.3	11.9	−4.9	4.5	1.5	1.3	1.1
	fatigue	12.2	32.6	−2.2	17.5	15.1	−0.4	9.1
**How does the virus spread to another person?**	by droplet transmission: coughing/sneezing	0.7	0.0	0.3	−1.3	−0.4	− 0.6	− 0.1
	by indirect contact: through items touched by the infected person	14.5	19.2	13.5	22.6	29.8	21.5	18.1
	by fecal-oral transmission: eating food contaminated with the virus	7.2	8.8	1.7	−0.9	6.7	−7.1	− 0.8
	by direct contact: touching infected person	6.5	9.3	2.8	7.4	18.0	18.9	9.2
**How to protect yourself from the virus?**	wear a standard (“surgical”) mask	58.0	46.9	41.1	51.2	51.3	37.4	44.8
	wear a mask with a HEPA filter	35.4	50.4	33.6	51.0	42.1	26.0	37.5
	wash your hands often	−0.8	−0.9	0.1	−1.1	0.0	0.7	− 0.3
	cover your mouth when coughing or sneezing	−2.1	−4.1	2.2	6.9	3.4	2.8	3.4
	keep at least 1 m distance from others.	−6.4	−2.8	5.8	4.1	1.1	5.8	3.4
	do not leave the house / flat	52.6	64.9	63.4	69.8	69.9	72.3	66.1
	contact the sanitary and epidemiological station after you came back from another country where Coronavirus disease 2019 has been reported	5.7	4.4	15.1	23.3	6.1	0.8	11.3
	drink alcohol	−0.5	−2.9	−2.6	−2.6	−3.6	−0.3	−2.3
	avoid products made in China	−1.2	−0.2	−1.9	−2.8	−2.8	−1.5	− 2.2
**What should I do if signs of infection appear?**	notify the sanitary and epidemiological station	0.3	1.1	6.8	5.1	8.6	11.1	6.6
	report to the emergency department in the nearest hospital	1.8	1.3	−4.6	−7.7	−12.2	−7.5	−6.4
	go to family doctor	0.9	−0.2	−2.4	−5.6	− 7.3	− 6.2	− 4.2
	report to an infectious disease ward or observation and infectious disease ward	−11.4	− 0.5	−26.0	− 6.4	− 26.4	− 21.3	− 19.1
**What would be for you the biggest obstacle in everyday life due to Coronavirus disease 2019 pandemic?**	inability to go to your workplace or place of study	− 18.0	− 10.7	14.5	3.4	9.7	13.0	8.2
	cancelled mass events, such as performances, conferences, concerts	1.8	6.3	−2.6	−4.0	4.1	3.2	−1.6
	cancelled trips, flights	6.4	4.7	−4.9	−0.7	6.3	−16.1	−4.1
	quarantine or isolation from loved ones due to quarantine	3.8	−15.5	15.0	−3.5	−3.3	17.9	5.6
	no products and food in shops or higher prices in stores	−40.2	−25.7	−34.5	− 32.9	− 43.7	− 45.0	−36.7
	no personal protection equipment in stock	12.0	11.1	6.7	8.1	4.3	−8.8	5.6
**Does any of the following aspects worries you due to the spreading infection?**	nothing worries me	−10.2	−5.9	−27.1	−23.0	−15.0	− 19.7	−19.6
	my own health and life	1.9	3.6	10.7	10.7	−1.4	5.6	5.9
	health and life of my family	19.9	12.6	34.3	28.1	21.0	22.9	26.5
	being quarantined	−14.4	−11.5	−16.8	− 18.9	−26.9	−15.9	− 18.2
	economic crisis	70.2	51.8	64.0	65.2	72.6	56.8	64.1
	my education	18.3	9.2	51.4	19.8	41.8	39.4	37.3
**Which of the following are your sources of knowledge about Coronavirus disease 2019?**	TV news	0.5	14.1	30.6	2.4	19.7	14.5	16.4
	radio	−3.0	0.5	6.3	−8.4	0.7	2.0	0.0
	newspapers	−0.2	6.5	1.0	1.3	−1.7	−1.3	0.1
	websites such as: onet, interia, wp, gazeta.pl, trójmiasto.pl	5.8	17.0	10.2	10.5	6.3	8.2	7.1
	website of the Ministry of Health of the Republic of Poland	10.9	16.0	27.7	28.0	31.8	22.3	27.4
	websites such as: onet.pl, interia.pl, wp.pl, gazeta.pl, trójmiasto.pl	4.0	8.8	0.3	22.5	18.3	−1.2	9.6
	family members and friends	2.0	5.6	−3.5	−6.8	7.5	13.4	0.2
	social media: Facebook, Tweeter, Instagram	−18.4	11.2	3.9	−6.1	−6.4	2.2	−3.6

## Discussion

The COVID-19 pandemic is a rapidly spreading global threat that in one way or another affects everyone, regardless of their profession and age. The study reported in this paper was a spontaneous and quick response to a unique and new social situation, designed to gain a measure of understanding of Poland’s population knowledge of and reactions at two very different phases of the epidemic’s development in Poland. Due to the crucial role of medical professionals in tackling the pandemic, the sample was divided into the two main cohorts, medical and non-medical.

### Communities’ knowledge about COVID-19

Even at the beginning of pandemic in Poland, nearly all of the respondents knew that the SARS-CoV-2 attacked the pulmonary system. Moreover, this level of knowledge was maintained a month later.

The three main symptoms of the infection (cough, fever, shortness of breath) were well known. This is similar to some studies [16–18], but different to other studies [19, 20]. Only the three main symptoms of the infection were shown on the governmental posters [21] and that is probably the reason why the non-medical respondents were mostly aware just of those. At the beginning of the epidemic, muscle pain and tiredness were mentioned only by specialised sources of scientific knowledge [1, 22] and that is probably the reason why only physicians knew about them**.** After a month, an overall increase in the awareness of other symptoms was observed.

In March 2020, Poles knew the COVID-19 was an airborne disease, similar to the UK and US populations [16]. After a month, they also knew that the virus could spread via direct and indirect contact. Researches marked answers pointing to these three ways of transmission as correct, as the preventive measures promoted by the government [21] to limit the spread of COVID-19 were: hand washing that limits indirect contact, covering mouth to reduce airborne transmission and social distancing, which reduces direct contact. People were highly aware of these prevention steps.

At the beginning of the epidemic, Poles knew they should call sanitary-epidemiological services or visit an infectious diseases ward if experiencing COVID-19 symptoms. Unfortunately, some people would also visit a general practitioner clinic or an emergency department. This would be a fundamental mistake as these places were not taking sufficient anti-COVID-19 preventive measures [23]. The same behaviour was noticed in South Korea during the MERS epidemics [24]. This is most likely due to the typical way the healthcare systems operate, including the Polish one. If one needs to see a physician, one goes to a general practitioner (mild cases) or an emergency department (severe cases). This changed during the pandemic as the healthcare system had to adapt to the new conditions. Fortunately, during the second assessment, a threefold and fourfold drop was observed in the percentage of respondents mentioning a general practitioner or emergency department visit with COVID-19 symptoms, respectively. This is an excellent improvement in the society’s knowledge that limits the COVID-19 spread. This happened as the “do not’s” were added on posters, too [25].

Why did the Poles have such high levels of knowledge about COVID-19 that further increased later on? At the beginning, the most crucial information (transmission routes, prevention, what to do if one has symptoms) was broadly disseminated via leaflets, posters [21, 25], and mass media news reports, and repeated multiple times so people had a chance to encounter and learn it. A similar pattern was observed in other countries affected by the pandemic [26]. What is more, before the virus arrived in Poland in March, the pandemic had already been ongoing for several weeks (months, if counting from the first Wuhan infections) and had already affected many other countries worldwide (China, South Korea) and in Europe (Italy) [3]. Thus, people in Poland had more time to prepare for the new threat. Searching for information using various media could be used to reduce anxiety levels by helping people to understand the upcoming, unprecedented situation [12, 26, 27]. On the practical level, increasing one’s knowledge helps to avoid infection [16].

The differences between medical and non-medical populations are understandable in the light of the requirement for continuous professional education among medical professionals. Furthermore, medical professionals form the first line of defence against COVID-19. Thus, this group had naturally more interest and a larger need to prepare early and more effectively to protect themselves.

### COVID-19 information sources and their evaluation

In general, people felt quite well informed to start with, and after a month the information confidence had slightly increased further. This is a result similar to other studies [18, 19, 28]. General news websites and social media were the main sources of knowledge for non-medicine-oriented respondents, which explains why at the beginning of the pandemic many people were not familiar with the up to date events and findings presented in scientific knowledge sources. Conversely, medicine-oriented community preferred science-oriented or governmental websites. Again, this group choose these sources to educate themselves as they were the first line of defence against COVID-19.

In the second wave of the study the respondents indicated using other sources of knowledge than they had used a month earlier. More respondents visited governmental and science-oriented websites. This difference was particularly noticeable among the non-medical cohort. Additionally, due to the lockdown restrictions and isolation [29], people were spending much more time at home. Thus, more people had time to watch television and learn about COVID-19 from TV broadcasts, which explains the greater popularity of this information source in the second round of the study.

### Pandemic-induced fears and their changes

At the beginning of the pandemic in Poland the virus had already spread to all inhabited continents and clearly affected societies worldwide. In general, insecurity was the most alarming factor for the Poles, who sometimes felt more threatened with COVID-19 compared to, for example, people in China [26]. The probable reason was that at the beginning of the epidemic in China, the residents were not aware of the gravity of the situation and the consequences that the pandemic would bring. Moreover, media were devoting a lot of coverage to the epidemic in China [30]. All these induced additional fears and psychological impact on the Poles and other nations [31].

A month after the first survey was conducted, the situation in Poland had changed dramatically. Numerous governmental restrictions were imposed on the citizens in order to stop COVID-19 from spreading [4–6]. As a result, more than 95% of the respondents surveyed in the second wave of the study were scared of something (picked at least one item in the 10th question) and a 20% increase in this measure was observed between the first and the second wave of the study (Tables 4 and 5).

More specifically, however, at the beginning people were worried about the quarantine (69.1%), rising grocery prices (61.2%), and their families (60.2%) (Table 2). Later on, the main fears included economic crisis (+ 64.1%), education system crisis (+ 37.3%), and families’ health (+ 26.5%). On the other hand, the respondents were less often afraid of quarantine (− 18.2%) or price increases (− 36.7%) (Table 5).

### The quarantine and isolation

As quarantine and isolation cause a separation from the family, loss of freedom, and boredom, it is understandable [27] that people would like to avoid it and that at the beginning of the epidemic they were scared of it. However, the quarantine and isolation is crucial in preventing the pandemic from spreading [1]. Due to their concerns quarantine and isolation some people might not comply with the lockdown rules, which could result in higher incidence rate in the population. After a month, the quarantine became widespread and many people themselves or their family members had to undergo quarantine. This familiarity may be the reason why, after a month of the lockdown, the respondents were less afraid of being quarantined.

### Worry about family members

At the beginning many people were worried about the health of their family, similarly to the results noted in other studies [28, 32, 33]. Such concerns increased as the government imposed physical distancing, quarantine and isolation, especially for the elderly who have the greatest mortality risk [29]. Over time, the number of cases of COVID-19 in Poland increased [8]. Additionally, many healthcare systems could not manage COVID-19 without being overwhelmed (lack of hospital beds and medical personnel) which resulted in a high death toll, for example in Italy [9]. These may be among possible reasons why concerns about the health of respondents’ families considerably increased and were the most frequently selected reason for worry in the second wave of the survey.

### Financial crisis

It was expected that the prices for products in shops would rise or be instable, as an effect of decreased and prolonged delivery services, access, utilization and stability [34, 35]. Such thinking caused many people to stockpile for the upcoming pandemic [36]. Moreover, a global economic recession is projected [37]. The fear of job loss is experienced by 26% of the Poles [38]. As a result, the threat of an economic crisis registered the greatest increase among all reasons for worry listed in the survey.

### Education crisis

Relatively low fear rate at the beginning of pandemic in three surveyed “student” cohorts was observed. With introduction of the lockdown restrictions, students could no longer attend their educational facilities. With time, students started to worry about their future and their fear rates increased in the wave two of the study. Similar trends are observed globally [14, 39, 40]. Students of medicine-oriented faculties reported this worry most frequently, likely because their courses included practical classes like laboratory classes or clinical workshops that cannot be taught online.

### Fears among medical professionals

Being the first line of defence against COVID-19 for over a month, there was no respondent among nurses and physicians who did not pick at least one reason for worry. This shows that as the pandemic spread, medical professionals felt more and more endangered, possibly due to rising stress levels and tiredness. Moreover, in the second wave of the study medical professionals were the only group who did not indicate their absence at work as a worrisome problem. There is a likelihood that they would rather not go to work if they had any choice. This interpretation is consistent with the results from other studies, where physicians reported reluctance to work and even considered resignation [41] or showed poorer mental health than the population’s average [18]. Moreover, doctors experience high levels of posttraumatic stress [42], which suggests a need to pay more attention to psychological problems of the medical professionals, especially as members of this population suffer from vicarious traumatization [12] and seek increased social support during the pandemic [43].

The significant advantage of this survey is the fact that the first phase of the survey was conducted at the onset of the pandemic in Poland [3]. This allowed for monitoring of the change in level of knowledge and attitudes to COVID-19 among the Poles by repeating the same survey after a month. Furthermore, an online survey is an optimal method for collecting data during this dynamically changing situation, since it is faster and requires no contact between people, thus being more convenient than traditional methods.

Due to the shortness of time available for creating an ad hoc spontaneous survey, the interpretation of the findings from the study was subject to methodological limitations. It should be mentioned that some facts about the virus have not yet been scientifically proven and they change over time. Moreover, the samples in wave one and wave two of the study were not matched, and their demographic and professional composition differed to some extent, as the researchers did not conduct a follow-up survey and, as a result, anyone could fill in the survey in either or both waves – thus it is possible that some of the responses in wave two were from respondents who completed wave one, and some responses were from new respondents. Regardless of this methodology limitation, the samples were treated as independent samples coming from the same population. To increase the likelihood of obtaining a comparable sample, the survey was distributed by the same information channels in both waves. It is also problematic to assess whether respondents looked up answers to any of the knowledge questions while completing an online questionnaire. In addition to that, the mean age of the sample was young, so in order to get wider and stronger results the survey should have included the whole adult population.

## Conclusions

Due to their good initial knowledge about symptoms, transmission, and preventive behaviours regarding COVID-19, the Polish population appears to have been prepared for the peak of pandemic’s cases. People had more short-term concerns, like the possibility of rising grocery prices or worries about coping with quarantine and isolation. Over time, the knowledge and the fears among the respondents changed and at the end of the study they reflected long-term pandemic management issues. After a month, more people reported being scared of the pandemic in general, as well as economic and healthcare crises. The quarantine was not perceived as being as stressful as often as in the first wave. Students concerns were related to the education crisis. Medical professionals reported higher level of fear of the pandemic than other groups included in the study. Over time, a transition from the use of popular mass-media to more scientific sources of information was observed among the non-medical sub-population.

Compared to other studies, this study used before-and-after approach, which highlights the changes in people’s knowledge and perception of the COVID-19 pandemic over a period of time and the progression of the pandemic. It shows Polish communities’ transition between the onset of the epidemic and the time of the most extreme lockdown provisions against the COVID-19 pandemic in Poland.

## Supplementary Information

**Additional file 1.**

## Data Availability

The datasets used and/or analysed during the current study are available from the corresponding author on reasonable request.

## Abbreviations

COVID-19: Coronavirus disease 2019
WHO: World Health Organisation
SARS-CoV-2: Novel coronavirus 2019-nCoV
