# Supplementary material for: Before-and-after online community survey on knowledge and perception of COVID-19 pandemic
Source: BMC Infect Dis. 2020 Nov 19;20:861. doi: 10.1186/s12879-020-05602-6 (PMC7675397; doi:10.1186/s12879-020-05602-6)
Supplement: Supplementary file 1 — Additional file 1. [file 12879_2020_5602_MOESM1_ESM.pdf]

The questionnaire

1. I am:

- a doctor
- a nurse
- a person with another profession
- a student of a medical faculty
- a student of a non-medical faculty
- a high school student

2. How old are you? …………..

3. Current place of residence: ………..

4. Which human organ system is usually infected by Coronavirus? (mark one correct answer)?

- digestive system
- muscular system
- nervous system
- respiratory system
- genitourinary system

5. What are the symptoms of Coronavirus disease 2019?

- fever
- cough
- breathlessness or breathing problems
- muscle pain
- fatigue

6. How does the virus spread to another person?

- by droplet transmission: coughing/sneezing

- by indirect contact: through items touched by the infected person
- by fecal-oral transmission: eating food contaminated with the virus
- by direct contact: touching infected person

7. How to protect yourself from the virus?

- wear a standard ("surgical") mask
- wear a mask with a HEPA filter
- wash your hands often
- cover your mouth when coughing or sneezing
- keep at least 1-metre distance from others.
- do not leave the house / flat
- contact the sanitary and epidemiological station after you came back from another country where Coronavirus disease 2019 has been reported
- drink alcohol
- avoid products made in China

8. What should I do if signs of infection appear?

- notify the sanitary and epidemiological station
- report to the emergency department in the nearest hospital
- go to family doctor
- report to an infectious disease ward or observation and infectious disease ward

9. What is your biggest obstacle in everyday life due to Coronavirus disease 2019 pandemic?

- inability to go to your workplace or place of study
- cancelled mass events, such as performances, conferences, concerts
- cancelled trips, flights

- quarantine/isolation from loved ones due to quarantine
- no products and food in shops or higher prices in stores
- no personal protection equipment in stock

10. Does any of the following aspects worries you due to the spreading infection?

- nothing worries me
- my own health and life
- health and life of my family
- being quarantined
- economic crisis
- my education

11. Which of the following are your sources of knowledge about Coronavirus disease 2019?

- TV news
- radio
- newspapers
- websites such as: onet.pl, interia.pl, wp.pl, gazeta.pl, trójmiasto.pl
- website of the Ministry of Health of the Republic of Poland
- websites of WHO, medycyna praktyczna, scientific research
- family members and friends
- social media: Facebook, Tweeter, Instagram

12. Do you feel sufficiently informed about the epidemic? (Scale from 1 to 5, where 1 means “not sufficiently informed” and 5 means “sufficiently informed”
